# Supplementary material for: Intracellular ATP levels in mouse cortical excitatory neurons varies with sleep–wake states
Source: Commun Biol. 2020 Sep 7;3:491. doi: 10.1038/s42003-020-01215-6 (PMC7477120; doi:10.1038/s42003-020-01215-6)
Supplement: Supplementary file 1 — Supplementary Information [file 42003_2020_1215_MOESM1_ESM.pdf]

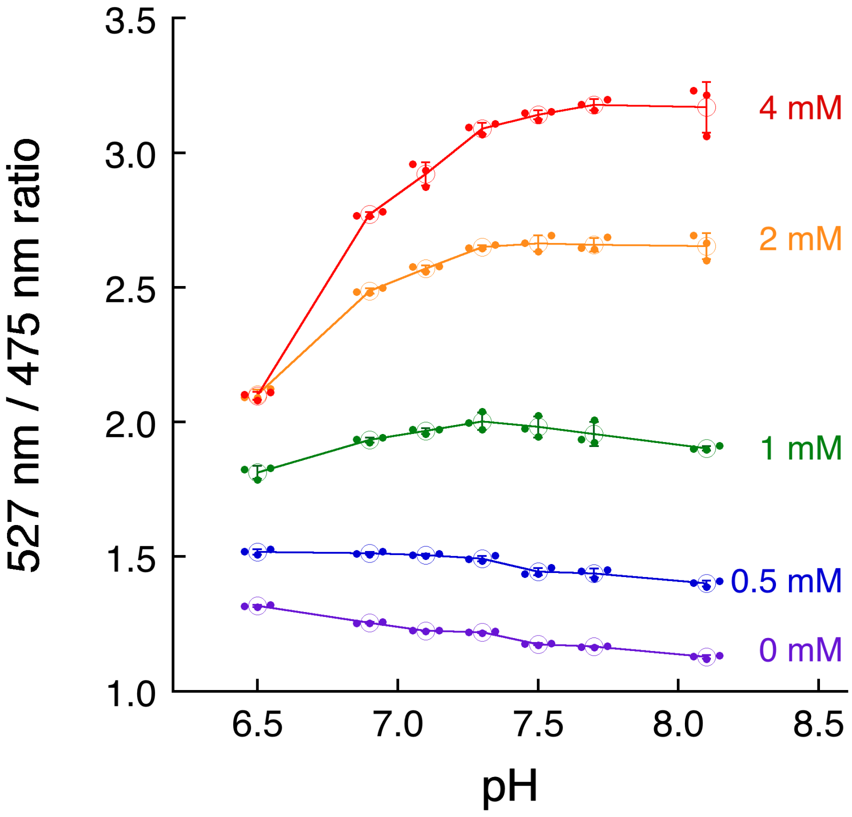

Supplementary Fig. 1 pH dependence of AT1.03^{YEMK}.

The fluorescence ratios (527/475 nm) of purified AT1.03^{YEMK} constructs at 37°C at 0, 0.5, 1, 2, and 4 mM ATP in the pH range of 6.5–8.1 are shown. The buffer contained 50 mM Mops-KOH (pH 6.5–7.5) or Hepes-KOH (pH 7.7–8.1), 50 mM potassium chloride, 0.5 mM magnesium chloride, and 0.05% Triton X-100. Data are presented as mean ± SD (n = 3 trials).

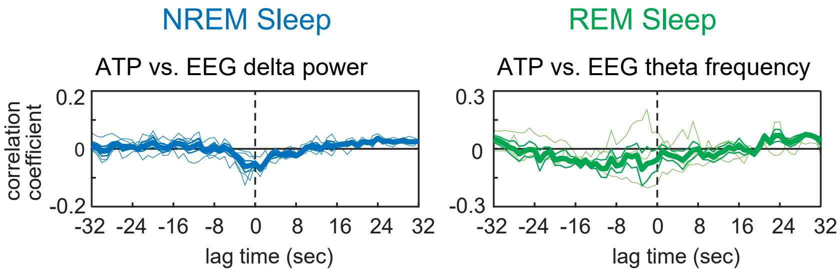

Supplementary Fig. 2 Temporal correlation between spontaneous intracellular ATP changes in cortical neurons and EEG parameters (related to Fig. 2).

Temporal correlation between spontaneous intracellular ATP dynamics and EEG delta power in NREM sleep and EEG theta frequency in REM sleep, respectively. The average correlation values (every 1 s, mean ± SEM: thick and thin lines, respectively) are plotted against time lag ($n = 5$ mice).

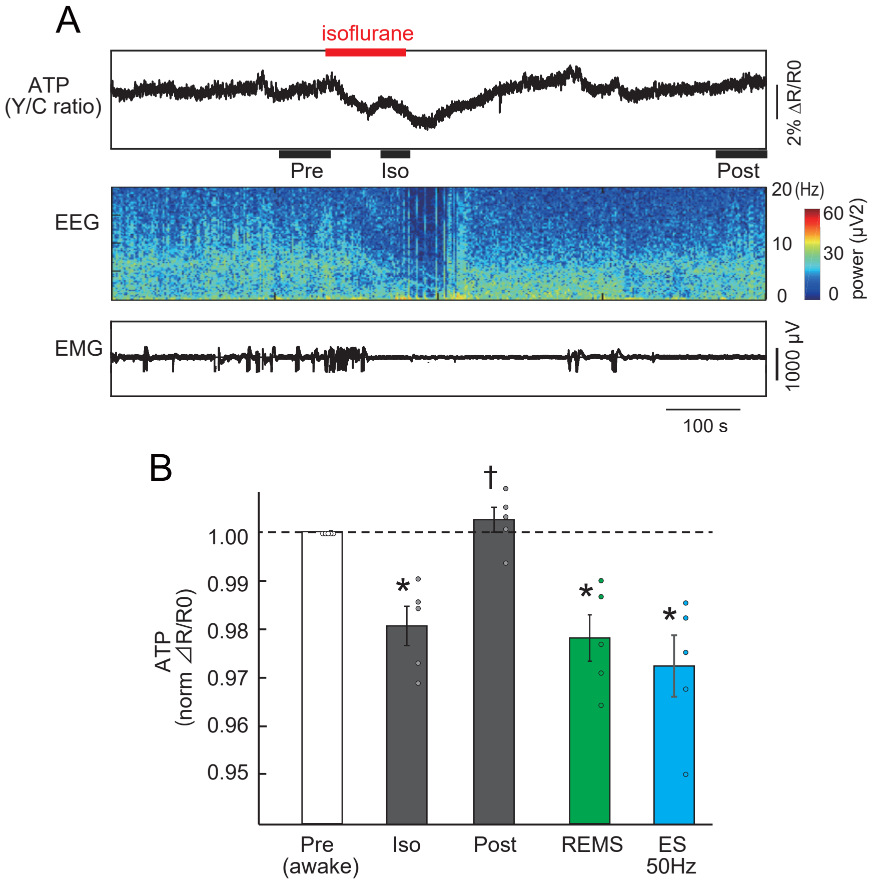

Supplementary Fig. 3 Intracellular ATP levels in cortical neurons in response to anesthesia (related to Fig. 2).

(A) Representative intracellular ATP signals (Y/C ratio) in cortical neurons, EEG and EMG signals during 2.5% isoflurane anesthesia. (B) ATP signals are suppressed during isoflurane anesthesia and restored thereafter. “Pre (awake)”: from –60 s to 0 s, “Iso”: from 71 s to 100 s, “Post”: from 841 s to 900 s (0 s: onset of isoflurane treatment). Data of “REMS” and “ES 50Hz” are trough values under these conditions from the same animals. Data are normalized to the mean value of Pre. *$p < 0.05$ vs. Pre and † $p < 0.05$ vs. Iso, two-way ANOVA followed by the Bonferroni post-hoc test ($n = 5$ mice). Data are presented as mean ± SEM.

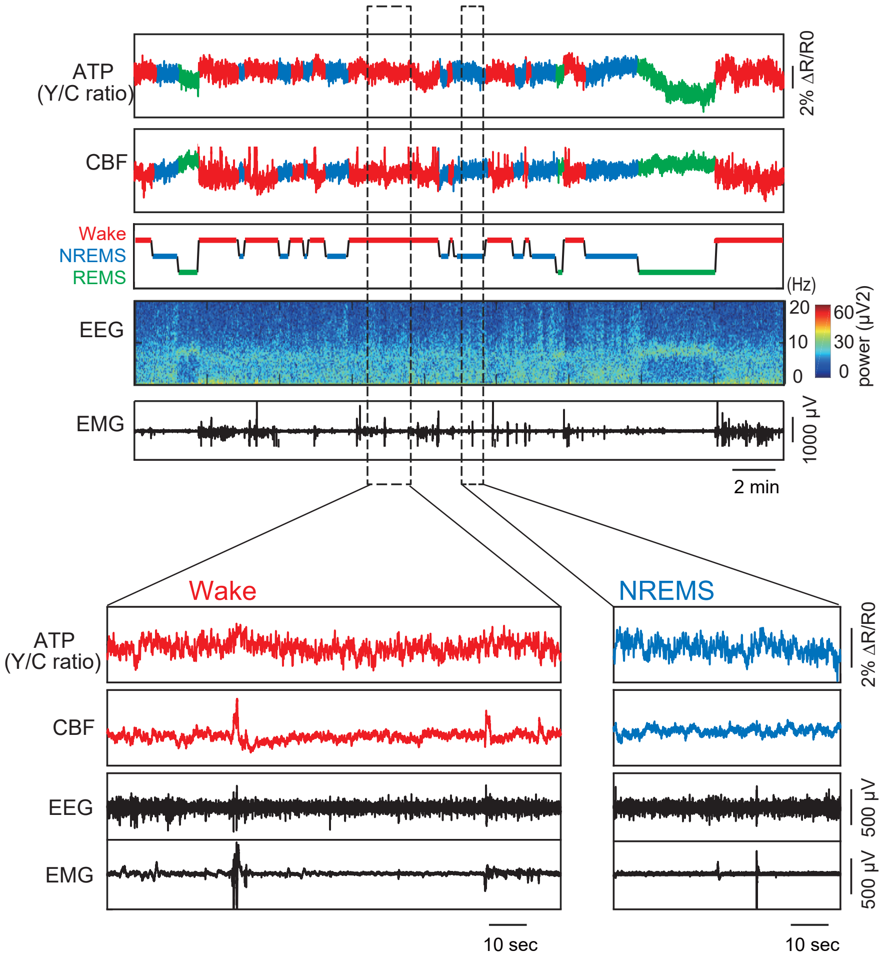

Supplementary Fig. 4 Physiological fluctuations of intracellular ATP levels in cortical neurons within the sleep-wake states (related to Fig. 3).

Example traces of fluctuations of intracellular ATP levels in cortical neurons and CBF accompanied by the change in EEG and EMG activities during the wake or NREM sleep (NREMS) states. The dotted box in the top panel (same as Fig. 2C) corresponds to the time interval shown enlarged in the bottom trace.

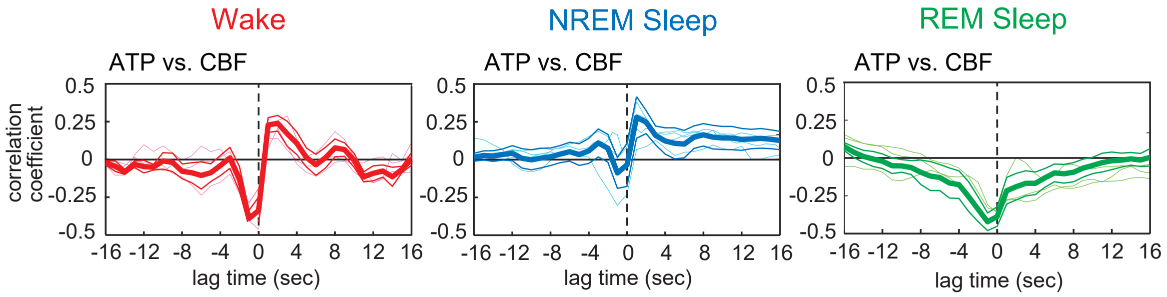

Supplementary Fig. 5 Temporal correlation between spontaneous intracellular ATP changes in cortical neurons and CBF (related to Fig. 3).

Temporal correlation between spontaneous intracellular ATP dynamics and CBF during the wake, NREM sleep, and REM sleep states, respectively. The average correlation values (every 1 s, mean ± SEM: thick and thin lines, respectively) are plotted against the time lag ($n = 5$ mice).

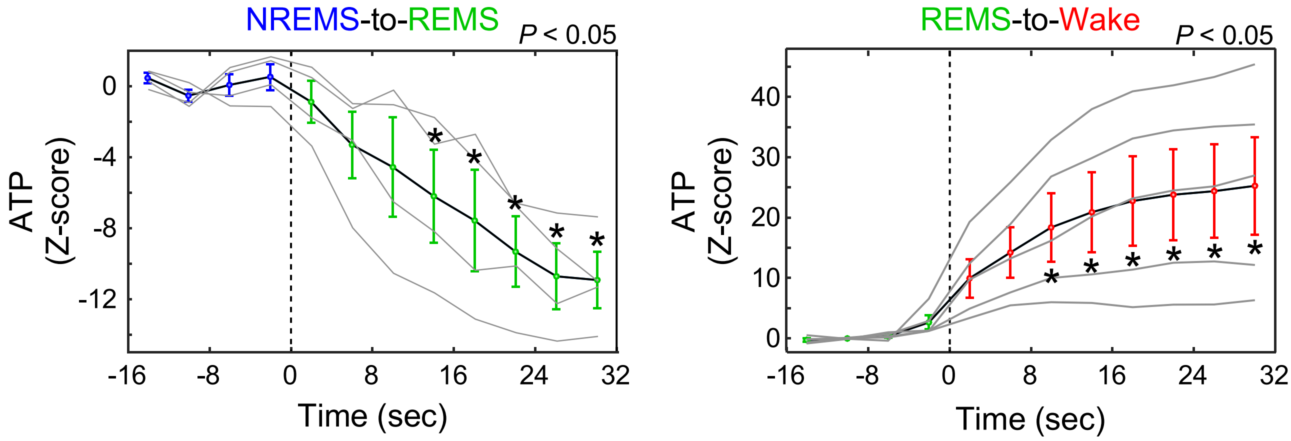

Supplementary Fig. 6 Global intracellular ATP changes in cortical neurons during state transitions (related to Fig. 4).

Mean ATP signals in whole cortical areas observed during state transitions of NREMS-to-REMS and REMS-to-wake. Transitions occurred at 0 s. Data are from 4-s intervals characterized by state transitions. ATP signals are averaged across the transitions exhibited by each animal. Group mean ± SEM of these averaged values are shown (n = 4 mice). P-values denote significant differences between states (two-way ANOVA with the Bonferroni post-hoc test). *$p < 0.05$ vs. first epoch before state transition.
