# Supplementary material for: Intracellular ATP levels in mouse cortical excitatory neurons varies with sleep–wake states
Source: Commun Biol. 2020 Sep 7;3:491. doi: 10.1038/s42003-020-01215-6 (PMC7477120; doi:10.1038/s42003-020-01215-6)
Supplement: Supplementary file 2 — Descriptions of Additional Supplementary Files [file 42003_2020_1215_MOESM2_ESM.pdf]

Descriptions of Additional Supplementary Files

File name: Supplementary Data 1
The source data for graphs in the figures.

File name: Supplementary Movie 1
Representative wide-field ATP imaging of the cortical surface of a Thy1-ATeam mouse during the transition from REM sleep to the wake state (related to Fig. 4). Scale bar = 1 cm.

File name: Supplementary Movie 2
Representative wide-field ATP imaging of the cortical surface of a Thy1-ATeam mouse during the transition from REM sleep to non-REM sleep (related to Fig. 4). Scale bar = 1 cm.

File name: Supplementary Movie 3
Representative wide-field ATP imaging of the cortical surface of a Thy1-ATeam mouse during the transition from non-REM sleep to REM sleep (related to Fig. 4). Scale bar = 1 cm.
