# Supplementary material for: Intracellular ATP levels in mouse cortical excitatory neurons varies with sleep–wake states
Source: Commun Biol. 2020 Sep 7;3:491. doi: 10.1038/s42003-020-01215-6 (PMC7477120; doi:10.1038/s42003-020-01215-6)
Supplement: Supplementary file 7 — Reporting Summary [file 42003_2020_1215_MOESM7_ESM.pdf]

nature research

Corresponding author(s): Akiyo Natsubori

Last updated by author(s): May 1, 2020

# Reporting Summary

Nature Research wishes to improve the reproducibility of the work that we publish. This form provides structure for consistency and transparency in reporting. For further information on Nature Research policies, see Authors & Referees and the Editorial Policy Checklist.

## Statistics

For all statistical analyses, confirm that the following items are present in the figure legend, table legend, main text, or Methods section.

| n/a | Confirmed | |
|---|---|---|
| ☐ | ☒ | The exact sample size (*n*) for each experimental group/condition, given as a discrete number and unit of measurement |
| ☐ | ☒ | A statement on whether measurements were taken from distinct samples or whether the same sample was measured repeatedly |
| ☐ | ☒ | The statistical test(s) used AND whether they are one- or two-sided<br>*Only common tests should be described solely by name; describe more complex techniques in the Methods section.* |
| ☐ | ☒ | A description of all covariates tested |
| ☐ | ☒ | A description of any assumptions or corrections, such as tests of normality and adjustment for multiple comparisons |
| ☐ | ☒ | A full description of the statistical parameters including central tendency (e.g. means) or other basic estimates (e.g. regression coefficient) AND variation (e.g. standard deviation) or associated estimates of uncertainty (e.g. confidence intervals) |
| ☐ | ☒ | For null hypothesis testing, the test statistic (e.g. *F*, *t*, *r*) with confidence intervals, effect sizes, degrees of freedom and *P* value noted<br>*Give P values as exact values whenever suitable.* |
| ☒ | ☐ | For Bayesian analysis, information on the choice of priors and Markov chain Monte Carlo settings |
| ☒ | ☐ | For hierarchical and complex designs, identification of the appropriate level for tests and full reporting of outcomes |
| ☒ | ☐ | Estimates of effect sizes (e.g. Cohen's *d*, Pearson's *r*), indicating how they were calculated |

*Our web collection on statistics for biologists contains articles on many of the points above.*

## Software and code

Policy information about availability of computer code

| | |
|---|---|
| Data collection | Each data collection procedures are described in the Online Methods. The fluorescence signals, EEG/EMG signals, hemodynamic signal and TTL signals were digitized by a data acquisition module (NI USB-6008, National Instruments), and simultaneously recorded using a custom-made LabVIEW program (National Instruments). Wide-field images were acquired using the HCImage Live software (Hamamatsu Photonics). Any data collection programs are available by sending a request to the corresponding author. |
| Data analysis | All offline computational analyses were performed using MATLAB (R2017b). Each analysis procedure is described in necessary detail within the "Data analysis" section of the Online Methods. Any analysis MATLAB codes are available by sending a request to the corresponding author. |

For manuscripts utilizing custom algorithms or software that are central to the research but not yet described in published literature, software must be made available to editors/reviewers. We strongly encourage code deposition in a community repository (e.g. GitHub). See the Nature Research guidelines for submitting code & software for further information.

## Data

Policy information about availability of data

All manuscripts must include a data availability statement. This statement should provide the following information, where applicable:

- Accession codes, unique identifiers, or web links for publicly available datasets
- A list of figures that have associated raw data
- A description of any restrictions on data availability

All the data are available from corresponding author upon suitable request .

# Field-specific reporting

Please select the one below that is the best fit for your research. If you are not sure, read the appropriate sections before making your selection.

☒ Life sciences ☐ Behavioural & social sciences ☐ Ecological, evolutionary & environmental sciences

For a reference copy of the document with all sections, see nature.com/documents/nr-reporting-summary-flat.pdf

# Life sciences study design

All studies must disclose on these points even when the disclosure is negative.

| | |
|---|---|
| Sample size | Sample sizes adequate for statistical tests (t-tests and one-way and two-way ANOVA) were determined, based on previous experiments from our laboratory and others. |
| Data exclusions | No data were excluded from the analyses. |
| Replication | Experiments were performed with sufficient animals to demonstrate statistical significance. All attempts at replication were successful. |
| Randomization | We used the mice for the experiments at random. |
| Blinding | The investigators were blinded to determinate the vigilance state of mice.<br>Another data collection and analysis were not performed blind to the conditions of the experiments. |

# Reporting for specific materials, systems and methods

We require information from authors about some types of materials, experimental systems and methods used in many studies. Here, indicate whether each material, system or method listed is relevant to your study. If you are not sure if a list item applies to your research, read the appropriate section before selecting a response.

## Materials & experimental systems

| n/a | Involved in the study | |
|---|---|---|
| ☒ | ☐ | Antibodies |
| ☒ | ☐ | Eukaryotic cell lines |
| ☒ | ☐ | Palaeontology |
| ☐ | ☒ | Animals and other organisms |
| ☒ | ☐ | Human research participants |
| ☒ | ☐ | Clinical data |

## Methods

| n/a | Involved in the study | |
|---|---|---|
| ☒ | ☐ | ChIP-seq |
| ☒ | ☐ | Flow cytometry |
| ☒ | ☐ | MRI-based neuroimaging |

## Animals and other organisms

Policy information about studies involving animals; ARRIVE guidelines recommended for reporting animal research

| | |
|---|---|
| Laboratory animals | Thy1-ATeam transgenic mouse line (Trevisiol, A. et al., 2017, eLife) was used. The genetic background was C57B6. Both male and female mice, 3- to 12-month-old were used for all experiments. |
| Wild animals | The study did not involve wild animals. |
| Field-collected samples | The study did not involve samples collected from the field. |
| Ethics oversight | All animal procedures were conducted in accordance with the National Institutes of Health Guide for the care and use of laboratory animals and approved by the Animal Research Committee of Tokyo Metropolitan Institute of Medical Science and Tohoku University. |

Note that full information on the approval of the study protocol must also be provided in the manuscript.
